# Restricted linear association between night sleep duration and diabetes risk in middle-aged and older adults: a 7-year follow-up analysis from the China health and retirement longitudinal study

**DOI:** 10.3389/fendo.2024.1364106

**Published:** 2024-06-20

**Authors:** Mutong Chen, Baizhi Li, Guanhua Fan, Yuqiu Zhou

**Affiliations:** ^1^ Health Management Center of Outpatient Department, Cancer Hospital of Shantou University Medical College, Shantou, China; ^2^ Shantou University Medical College, Shantou, China; ^3^ Nursing College, Harbin Medical University, Daqing, Heilongjiang, China

**Keywords:** older adults, night sleep duration, diabetes mellitus, non-linear association, Chinese population, CHARLS

## Abstract

**Background:**

A rapid increase in the prevalence of diabetes is an urgent public health concern among older adults, especially in developing countries such as China. Despite several studies on lifestyle factors causing diabetes, sleep, a key contributor, is understudied. Our study investigates the association between night sleep duration and diabetes onset over a 7-year follow-up to fill information gaps.

**Method:**

A population-based cohort study with 5437 respondents used 2011–2018 China Health and Retirement Longitudinal Study data. Using self-reported night sleep duration from the 2011 baseline survey, information on new-onset diabetes was collected in follow-up surveys. Baseline characteristics of participants with vs. without new-onset diabetes were compared using Chi-square and Mann-Whitney U tests. Multivariable Cox regression models estimated the independent relationship between night sleep and new-onset diabetes. The addictive Cox regression model approach and piece-wise regression described the nonlinear relationship between night sleep and new-onset diabetes. Subgroup analysis was also performed by age, gender, body measurement index, dyslipidemia, drinking status, smoking, hypertension, and afternoon napping duration.

**Result:**

549 respondents acquired diabetes during a median follow-up of 84 months. After controlling for confounders, night sleep duration was substantially linked with new-onset diabetes in the multivariable Cox regression model. The risk of diabetes is lower for respondents who sleep longer than 5 hours, except for those who sleep over 8 hours [5.1–6h Hazard ratios (HR) [95% confidence intervals (CI)] = 0.71 (0.55, 0.91); 6.1–7h HR = 0.69 (0.53, 0.89); 7.1–8h HR = 0.58 (0.45, 0.76)]. Nonlinear connections were delineated by significant inflection points at 3.5 and 7.5 hours, with a negative correlation observed only between these thresholds. With one hour more night sleep, the risk of diabetes drops 15%. BMI and dyslipidemia were identified as modifiers when only consider the stand linear effect of sleep duration on diabetes.

**Conclusion:**

This study establishes a robust association between night sleep and new-onset diabetes in middle-aged and older Chinese individuals within the 3.5–7.5-hour range, offering a foundation for early glycemic management interventions in this demographic. The findings also underscore the pivotal role of moderate night sleep in preventing diabetes, marking a crucial juncture in community medical research.

## 1 Introduction

The ever-expanding global public health concern of diabetes mellitus, coupled with its numerous comorbidities, has progressively captured the attention of the medical community (1). Over the antecedent decades, the adult populace grappling with diabetes has demonstrated a dramatic surge, particularly in countries with low to middle income such as China (2). According to the International Diabetes Federation, China carries the weighty mantle of possessing the highest diabetes-afflicted populace, exceeding 140 million sufferers as of 2021; a staggering figure anticipated to escalate beyond 174 million by the year 2045 (3). As known, type 2 diabetes is the predominant type of diabetes, accounting for approximately 90% of adult individuals with diabetes (4). Although genetic predisposition partially determines individual susceptibility to type 2 diabetes, the sedentary lifestyle and unhealthy diet serve as crucial driving factors of the current global epidemic (4, 5). Strong evidence suggests that a significant number of type 2 diabetes cases could be prevented by maintaining a healthy body weight, adhering to a healthy diet, engaging in at least 30 minutes of daily exercise, avoiding smoking, and practicing moderate alcohol (6, 7). Accordingly, identifying modifiable factors associated with diabetes and understanding their association can contribute to improving intervention strategies and alleviating the disease burden.

Adequate and high-quality night sleep is fundamentally indispensable for maintaining holistic physical and psychological health (8). With the inevitability of aging, however, comes an increased propensity for sleep irregularities, including an insufficiency or surplus of night sleep (9, 10). Sleep irregularity serves as a modifiable risk factor, which is considered as one of the most important causes of diabetes. Previous studies have indicated that new-onset diabetes is a prevalent affliction amongst middle-aged and older adults, with a close association with night sleep duration. The most comprehensive meta-analysis to date proposed a U-shaped relationship between night sleep duration and the incidence of Type 2 diabetes (11). Findings from a U.K. Biobank cohort analysis showed that individuals with unfavorable sleep and circadian patterns were at an elevated risk of developing Type 2 diabetes (12). In Liu’s Mendelian randomized study, the presence of insomnia traits or inadequate night sleep was causatively linked to elevated levels of HbA1c (13). When focusing specifically on the Chinese population, the association between nighttime sleep and diabetes remains valid, yet results are still inconsistent (10). For example, a prospective study from the Shanghai Men’s Health Study involving 34,825 participants found a slight increase in diabetes risk for those sleeping 8 hours or more compared to those sleeping 7 hours (HR = 1.2, 95% CI = 1.0–1.3) (14). Another study of 11,539 Chinese participants with three years follow-up observed higher diabetes risk in those sleeping over 9 hours, with no significant difference in those sleeping less than 6 hours compared to 7–8 hours (10). Though these studies unanimously indicate a nonlinear relationship between nighttime sleep and diabetes, inconsistencies in their results strongly reduce their practical relevance. Therefore, further study for the Chinese population using a representative cohort with a sufficient follow-up time is still required.

In this study, we utilized the latest 2018 follow-up data from China Health and Retirement Longitudinal Study (CHARLS) to assess the relationship between night sleep duration and the new-onset of diabetes among Chinese middle-aged and older individuals. Moreover, we employed non-linear methods to elucidate the association between night sleep duration and newly diagnosed diabetes. Our research aimed to lay the groundwork for devising early intervention strategies for glucose management in middle-aged and older populations, as an integral part of community healthcare practices.

## 2 Method

### 2.1 Study design and participants

The current cohort study embodies a secondary analysis of data originating from the CHARLS, an ongoing, nationally representative longitudinal investigation (15). The extensive details of the study design have been delineated in a separate publication (15). In summary, a total of 17,708 participants across 10,257 households were recruited between June 2011 and March 2012 from 150 distinct counties or districts, encompassing 450 villages spanning 28 provinces within China. The recruitment strategy was based on the application of a multistage stratified probability-proportional-to-size sampling technique. Household with a member 39 years of age or older was randomly selected, and at least one of member aged in 39 or older will be selected as study sample. If the selected person was between 39 and 45 years of age that person was designated for inclusion in a future refreshment sample and was not interviewed. If the chosen person was 45 years of age or older, both that person and his or her spouse were interviewed. Therefore, we only included those who were at least 45 years old at the first wave as our study sample. Participants undertook the completion of a standardized questionnaire aimed at capturing a diverse array of sociodemographic, lifestyle factors, and health-associated details. An impressive 80.5% response rate was achieved in the initial survey. Physical activity is acquired in a subsample of half the sample, using the modified International Physical Activity Questionnaire-short form. Subsequent follow-ups were planned biennially post the baseline survey. The CHARLS received the necessary ethical approval from the Institutional Review Board of Peking University and written informed consent was obtained from all participants. The present study adhered to the guidelines stipulated under the Strengthening the Reporting of Observational Studies in Epidemiology (STROBE) (16).

### 2.2 Study population

**Figure 1:**
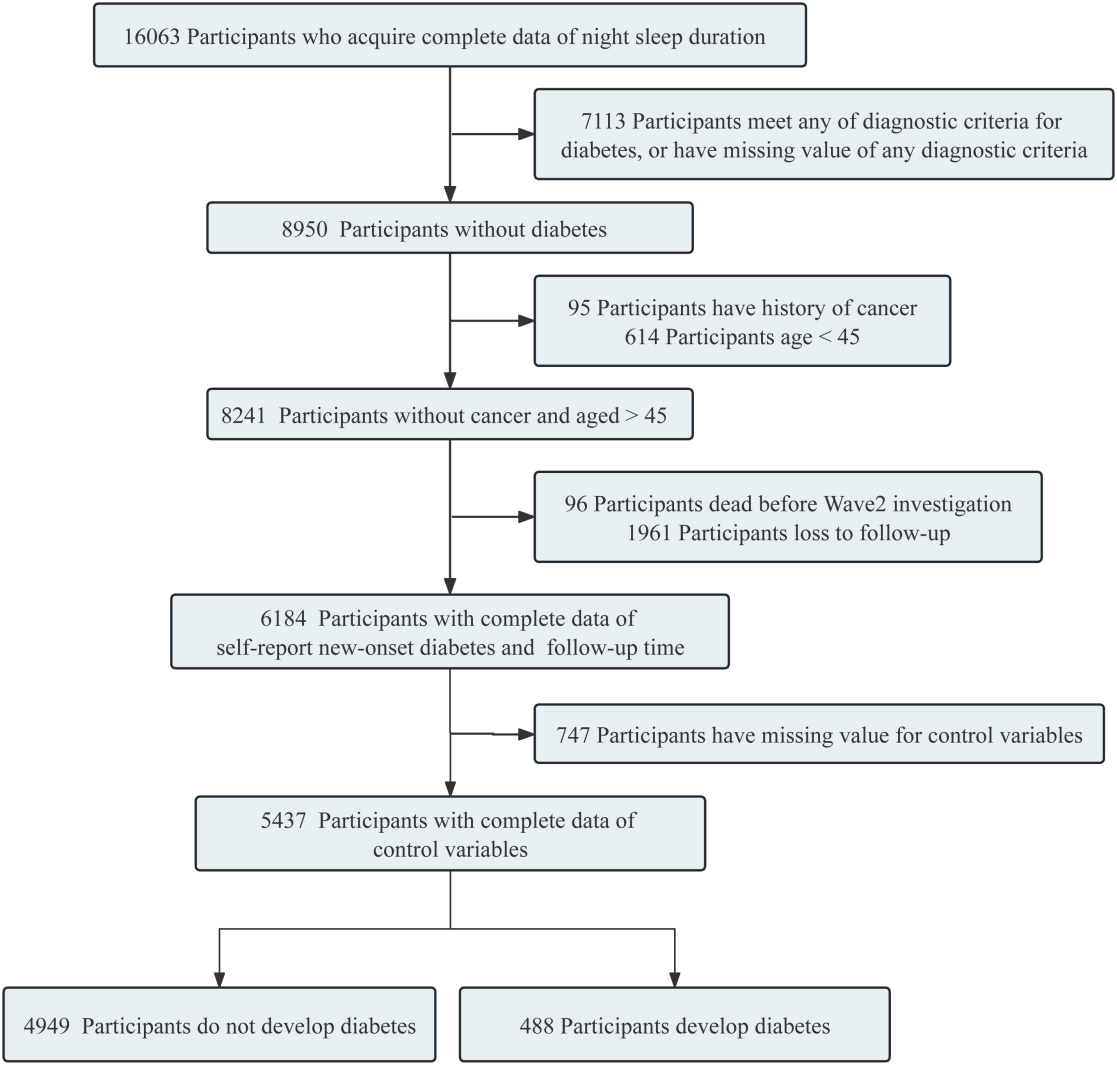
Flow chart of inclusion and exclusion process.

The analysis began with the inclusion of the data from 16,063 participants with complete baseline responses (W1, 2011) on night sleep duration. Next, individual meeting the relevant exclusion criteria were further excluded. Exclusion criteria comprised individuals 1) in the W1 baseline survey who satisfied any of the diagnostic criteria for diabetes (17) (namely, fasting plasma glucose levels ≥ 126 mg/dL [converted to mmol/L by multiplying by 0.0555], HbA1c ≥ 6.5%, current usage of modern antidiabetic medications, or self-reported history of diabetes), 2) with missing data for any of the diabetes diagnostic criteria, 3) with a self-reported history of cancer, and 4) aged 45 years or younger, 5) documented deaths before diabetes incidence or missing follow-up information, 6) with missing data in any control variables. The remaining pool of 5437 participants’ data was included for analysis: among them, 488 developed diabetes during follow-up. A detailed participant enrollment flowchart is illustrated in **Figure 1**, providing a comprehensive visual overview of the recruitment process.

### 2.3 Conceptual framework of current study

**Figure 2:**
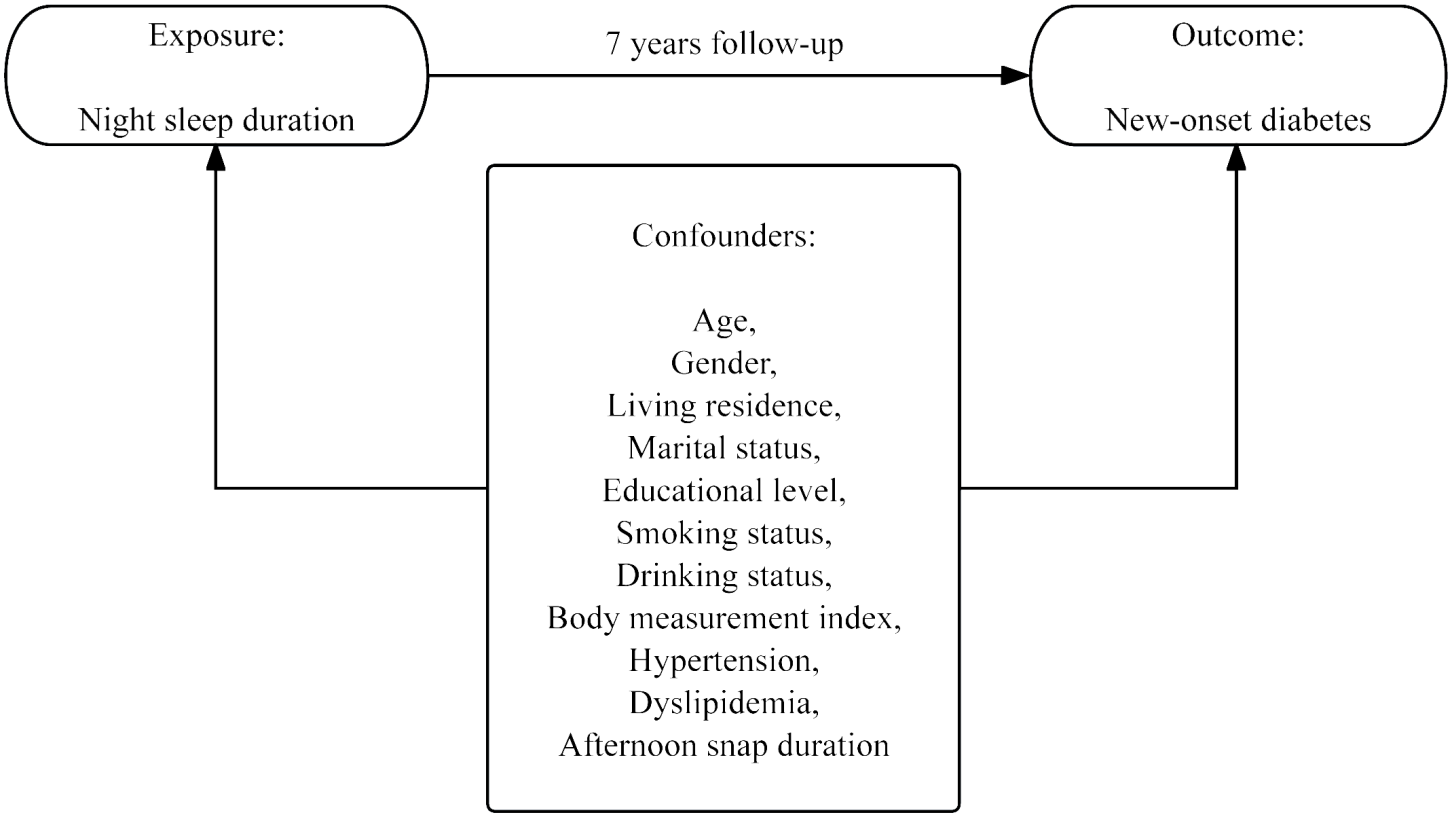
Conceptual framework for this study.

**Figure 2** depicts the proposed conceptual framework for the current study. The exposure variable is the duration of night sleep at baseline, and the outcome variable is new-onset diabetes throughout a 7-year period. The supposed casual effects are based on the temporal order of exposure and outcome variables. Potential confounders are considered because of their synergistic relationship with exposure and outcome variables, including age, gender, education level, marital status, living residence, smoking status, drinking status, BMI, hypertension, dyslipidemia, and afternoon snap sleep duration. Abbreviations: BMI, body mass index.

#### 2.3.1 Identification of new-onset diabetes (outcome)

New-onset diabetes was characterized by an affirmative response to the question: “Have you received a diagnosis of diabetes or high blood sugar?” The person-time follow-up for each participant was computed from the baseline survey date to the date of diabetes diagnosis or the participant’s date of death.

#### 2.3.2 Assessment of night sleep duration (exposure)

We evaluated night sleep duration based on responses to the question: “In the past month, how many hours of actual sleep did you get on an average night? (Note, this may be less than the total number of hours you spent in bed.)” Based on the sleep patterns observed within the participant pool, responses were arranged into five groups: ≤5 hours, 5.1–6 hours, 6.1–7 hours, 7.1–8 hours, and >8 hours. Additionally, to structure a regression model with a smooth term and price-wise model, we employed continuous night sleep duration data.

#### 2.3.3 Covariates (potential confounders)

The selection of covariates was informed by previous research and encompassed the following: age, gender, living residence, marital status, educational level, smoking and drinking status, body measurement index (BMI), hypertension, dyslipidemia, and afternoon snap duration (10, 18).

Trained interviewers collected sociodemographic and health-related information at baseline using a structured questionnaire. This encompassed details on age, sex, living residence (rural or urban), marital status (married or other [never married, separated, divorced, or widowed]), and educational level (formal education, primary school, middle or high school, or college or above). Health-related factors included self-reported smoking and drinking status, categorized into three levels: never, former, or current. A trained nurse undertook measurements of height, weight, and blood pressure.

BMI was computed as weight in kilograms divided by the square of height in meters, and classified into three levels: <18.5, 18.5–23.9, and ≥24 kg/m2. Hypertension was diagnosed if systolic blood pressure was ≥140 mm Hg, diastolic blood pressure was ≥90 mm Hg, if the participant was currently taking antihypertensive medications, or if they self-reported a history of hypertension. Dyslipidemia was defined as total cholesterol ≥240 mg/dL [converted to mmol/L by multiplying by 0.0259], triglycerides ≥150 mg/dL, low-density lipoprotein cholesterol ≥160 mg/dL, high-density lipoprotein cholesterol <40 mg/dL, current use of lipid-lowering medications, or self-reported history of dyslipidemia.

The afternoon napping duration was based on responses to the question: “Over the past month, on average, how long have your post-lunch naps been?” Guided by earlier research (19, 20) and the participants’ reported nap habits, the responses were allocated into four distinct categories: 0, <30 min, 31–90 min, and >90 min.

### 2.4 Statistical analysis

#### 2.4.1 Data representation and initial analysis

Continuous variables adhering to a normal distribution are represented as means and standard deviations (SDs), while categorical variables are expressed as frequencies and percentages. Baseline traits of participants, stratified based on new-onset diabetes, were summarized and analyzed using the chi-squared test and Mann-Whitney U test for comparison.

#### 2.4.2 Addressing missing data

Percentiles of missing values among covariates spanned from 0% to 11% (specifically, 8% and 473 observations for hypertension, 1% and 44 observations for dyslipidemia, 1 observation for drinking status, and 11% and 671 observations for BMI; the total observation count with all variables was 5437). In the primary analysis, instances of incomplete observations were omitted based on variables adjusted within the model.

#### 2.4.3 Application of cox proportional hazards models

Hazard ratios (HRs) and 95% confidence intervals (CIs) were calculated to investigate the association between night sleep duration and new-onset diabetes by employing Cox proportional hazards models. The proportional hazards assumption was tested with Schoenfeld’s global test and found no violation. Two models were established to examine the association between night sleep duration and new-onset diabetes.

#### 2.4.4 Model formulation

Model 1 was the crude model, with no adjustments for covariates.

Model 2 is the full model, which included adjustments for age, sex, education, marriage, living residence, smoking status, drinking status, BMI, hypertension, dyslipidemia, and daytime sleep duration.

#### 2.4.5 Exploration of non-linear trends, stability analysis and interaction test

To validate the potential non-linear association between night sleep duration and new-onset diabetes during 7-year follow-up, the additive Cox model was employed for curve-fitting method. Once non-linear trend was observed from the figure result of additive Cox model, a recursive algorithm (identifying the inflection point where the piecewise Cox has the largest maximum likelihood value using recursive method) was used to identifying inflection points.

Piecewise Cox model was constructed based on inflection points, and log-likelihood ratio test was used to test the difference between the piecewise model and one-line (non-segmented) model. To exploit whether this non-linear association remains stable in different stratification of given variable, we also conducted stratified analysis. Meanwhile, P-values for interaction were calculated by comparing models with or without interaction terms of night sleep duration and potential effect modifiers using likelihood ratio tests.

Three sensitivity analyses were performed to ensure the robustness of our results. In the first sensitivity analysis, we further considered sleep quality as a covariate. An evaluation question from Center for Epidemiologic Studies Short Depression Scale 10 (CESD-10) scale was used for evaluating the sleep quality: “Did you feel your sleep was restless during the past week?”. The following response options were provided: “Rarely or none of the time <1 day”, “some or a little of the time 1–2 days”, “occasionally or a moderate amount of 3” and “Most or all of the time 5–7 days”.

We further adjust this variable in our full model and serve it as the first sensitivity analysis., Given the different effects of moderate vs. heavy drinking on the diabetes risk in previous studies, we replaced the original drinking status with drinking among in the full model as the second sensitivity analysis. Drinking amount was categorized to non-drinker vs. moderate (≤1 drink/d for females, ≤2 drinks/d for males) vs. heavy (>1 drink/d for females, >2 drinks/d for males). In the third sensitivity analysis, missing values in covariates were assumed to be missing completely at random (MCAR), and multiple imputation method was used to generate 5 post-interpolation datasets which will be used for re-generating previous adjusted models.

Statistical significance was set at a two-sided P value of <0.05. All analyses were executed using R version 4.2.1 (R Foundation).

## 3 Results

### 3.1 Participant characteristics and diabetes incidence

**Table 1** encapsulates the demographic and health characteristics of the analyzed participants, which have been systematically stratified according to whether they had been diagnosed with diabetes during the follow-up period. The mean age of the participants was found to be 58.80 years, with a standard deviation (SD) of 8.55.

**Table 1:** Basic characteristics of the analyzed participants grouped by new-onset diabetes.

Characteristics	Having new-onset diabetes			
	Overall	No	Yes	P-value
Total Participants(n)	5437	4949	488	
Night sleep duration [mean (SD)]	6.34 (1.89)	6.37 (1.89)	6.07 (1.91)	**0.001**
Night sleep duration (discontinuous) (%)				**<0.001**
≤5	1610 (29.61)	1422 (28.73)	188 (38.52)	
>8	441 (8.11)	403 (8.14)	38 (7.79)	
5.1–6	1137 (20.91)	1043 (21.07)	94 (19.26)	
6.1–7	1055 (19.40)	970 (19.60)	85 (17.42)	
7.1–8	1194 (21.96)	1111 (22.45)	83 (17.01)	
Afternoon snap duration (discontinuous) (%)				0.291
0min	2659 (48.91)	2433 (49.16)	226 (46.31)	0.641
≤30min	876 (16.11)	790 (15.96)	86 (17.62)	
31–90min	1315 (24.19)	1193 (24.11)	122 (25.00)	
>90min	587 (10.80)	533 (10.77)	54 (11.07)	
Age [mean (SD)]	58.80 (8.55)	58.73 (8.55)	59.51 (8.48)	0.054
Gender = Male (%)	2489 (45.78)	2300 (46.47)	189 (38.73)	**0.001**
Education level (%)				0.264
No formal education	2609 (47.99)	2354 (47.57)	255 (52.25)	
primary school	1246 (22.92)	1145 (23.14)	101 (20.70)	
Middle school or high school	1445 (26.58)	1324 (26.75)	121 (24.80)	
College or above	137 (2.52)	126 (2.55)	11 (2.25)	
Marital status = Married or partnered (%)	4856 (89.31)	4430 (89.51)	426 (87.30)	0.151
Living residence = Urban (%)	1743 (32.06)	1580 (31.93)	163 (33.40)	0.538
Smoking status (%)				**0.01**
never	3337 (61.38)	3005 (60.72)	332 (68.03)	**0.004**
former	436 (8.02)	398 (8.04)	38 (7.79)	
now	1664 (30.61)	1546 (31.24)	118 (24.18)	
Drinking status (%)				0.053
never	3360 (61.80)	3041 (61.45)	319 (65.37)	
former	429 (7.89)	385 (7.78)	44 (9.02)	
now	1648 (30.31)	1523 (30.77)	125 (25.61)	
BMI (%)				**<0.001**
<18.5	367 (6.75)	338 (6.83)	29 (5.94)	
18.5–23.9	2966 (54.55)	2767 (55.91)	199 (40.78)	
>=24	2104 (38.70)	1844 (37.26)	260 (53.28)	
Hypertension (%)				**<0.001**
No	3337 (61.38)	3095 (62.54)	242 (49.59)	
Yes	2100 (38.62)	1854 (37.46)	246 (50.41)	
Dyslipidemia (%)				**<0.001**
No	2996 (55.10)	2796 (56.50)	200 (40.98)	
Yes	2441 (44.90)	2153 (43.50)	288 (59.02)	

Data are presented as mean (SD) or n (%).

Bold P values are significant at P < 0.05.

BMI, body mass index; HA1c, glycosylated hemoglobin; TC, total cholesterol; HDL, high-density lipoprotein; LDL, low- density lipoprotein; TG, triglyceride; SD, standard deviation.

The educational attainment was relatively low, with approximately half of the participants (47.99%) not having received formal education. Most participants were married or partnered (89.31%), lived in a rural area (67.94%), and were nonsmokers (61.38%) and nondrinkers (61.80%). A significant proportion of the cohort had a BMI within the average range (54.55%).

Participants reported an average night sleep duration of 6.34 hours (SD = 1.89), and 48.91% stated that they did not take afternoon snaps. In exploring the relationship between sleep patterns and the onset of diabetes, the study revealed that the mean night sleep duration was higher in the participants without new-onset diabetes (6.37 hours) compared to those with it (6.07 hours, P < 0.001). A trend was observed wherein participants with new-onset diabetes were more likely to be female, possess less formal education smoke and drink less, have a higher BMI, and have hypertension and dyslipidemia. Metabolic markers tested in the wave 1 also presented in **Supplementary Table 1**.

Compared with the group who do not have new-onset diabetes, participants who have new-onset diabetes during the 7 years follow-up had a higher levels of HbA1c (5.24 vs 5.09, P < 0.001), fasting glucose (105.02 vs 99.56 mg/dl, P < 0.001), total cholesterol (197.46 vs 192.34 mg/dl, P = 0.004), low-density lipoprotein (120.51 vs 117.10 mg/dl, P = 0.034), triacylglycerol (141.47 vs 119.32 mg/dl, P <0.001), a lower levels of high-density lipoprotein (49.06 vs 52.55 mg/dl, P < 0.001).

### 3.2 The association between night sleep duration, daytime sleep duration and new-onset diabetes

**Table 2** presents the outcomes from the regression investigations. Model 1, when benchmarked against a night sleep duration of less than 5 hours, divulged HRs (and their 95% CIs) for new-onset diabetes as follows: 0.7 (0.54, 0.89) for 5.1–6 hours, 0.66 (0.51, 0.86) for 6.1–7 hours, 0.57 (0.44, 0.73) for 7.1–8 hours, and 0.7 (0.49, 0.99) for sleep duration exceeding 8 hours. These associations, even after adjusting for aspects such as afternoon snap duration, age, sex, education, marital status, geographical residence, lifestyle factors, BMI, hypertension, and dyslipidemia, remained significant in all models barring the group that sleeps more than 8-night hours (HR = 0.71, 95% CI: [0.50, 1.01]).

**Table 2:** Associations of night sleep duration (discontinuous) with new-onset diabetes in Chinese middle-aged and older adults.

Characteristic	N	Event N	HR	95% CI	P-value
Model 1					
Night sleep duration					
<=5	1,610	188	Reference		
>8	441	38	0.7	0.49, 0.99	**0.043**
5.1–6	1,137	94	0.7	0.54, 0.89	**0.004**
6.1–7	1,055	85	0.66	0.51, 0.86	**0.002**
7.1–8	1,194	83	0.57	0.44, 0.73	**<0.001**
Model 2					
Night sleep duration					
<=5	1,610	188	Reference		
>8	441	38	0.71	0.50, 1.01	0.054
5.1–6	1,137	94	0.71	0.55, 0.91	**0.007**
6.1–7	1,055	85	0.68	0.53, 0.89	**0.004**
7.1–8	1,194	83	0.58	0.45, 0.75	**<0.001**

Model 1: Crude model; Model 2: Adjusted for age, sex, education, marital status, living residence, smoking status, drinking status, BMI, hypertension, and dyslipidemia, and afternoon snap duration.

Bold P values are significant at P < 0.05.

BMI, body mass index; HR, hazard ratio; CI, confidence interval.

**Figure 3:**
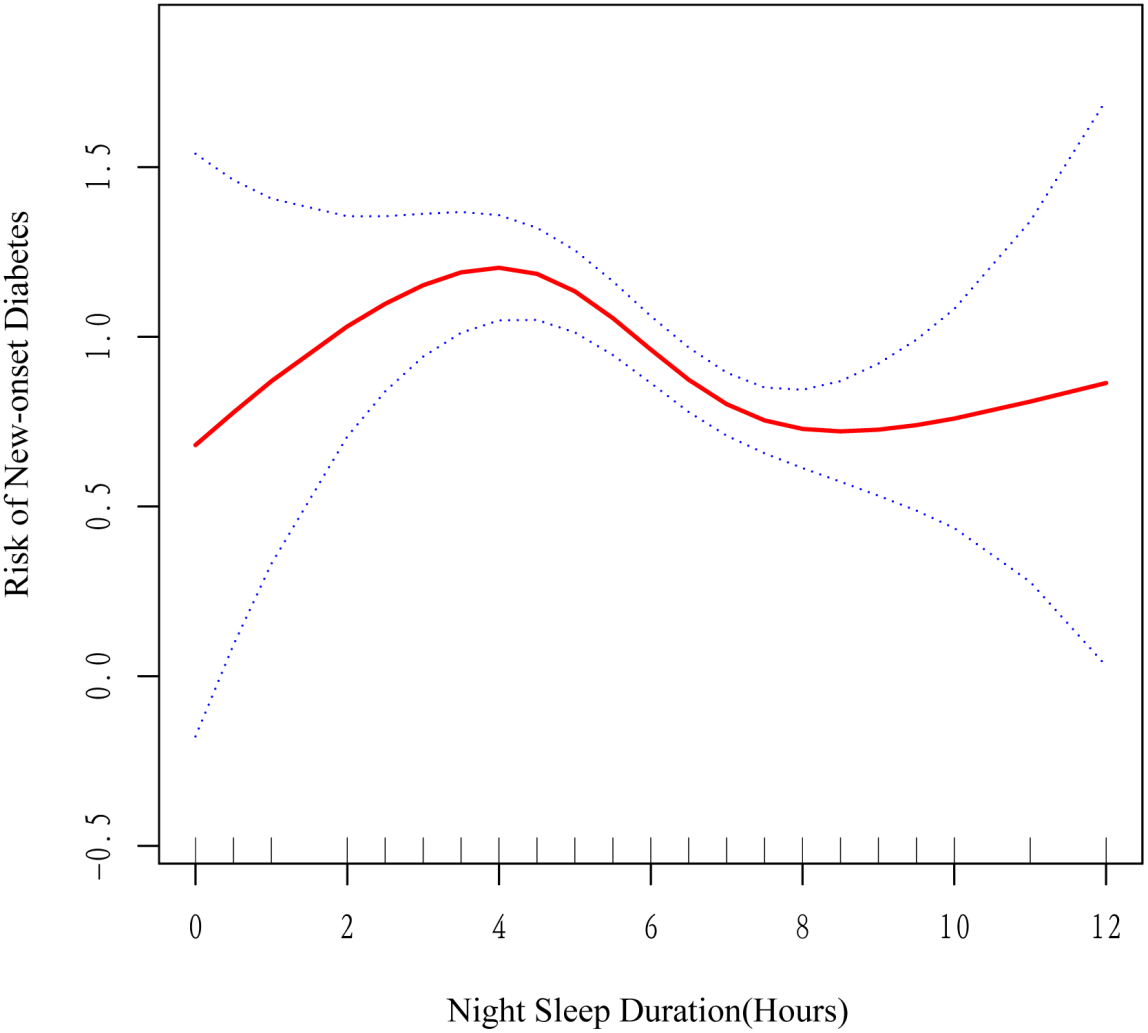
Non-linear association of night sleep duration with new-onset diabetes in Chinese middle-aged and older adults (The association was adjusted for age, gender, education level, marital status, living residence, smoking status, drinking status, BMI, hypertension, dyslipidemia, and afternoon snap sleep duration. Blue dash line represents the 95% CI from the fit. The solid red line represents the smooth curve between night sleep duration and the risk of new-onset diabetes. BMI, body mass index).

To explore the potential non-linear association, we employed addictive Cox regression model. As shown in **Figure 3**, after adjusting for age, gender, education level, marriage, living residence, smoking status, drinking status, BMI, hypertension, dyslipidemia, and afternoon napping duration, we discovered a fluctuated association between night sleep duration and new-onset diabetes. With sequential usage of recursive algorithm, we identified two inflection points, which are 3.5 and 7.5 hours. Based on these two inflection points, we combined a Cox model with a three-piecewise Cox model to fit this nonlinear association (P for log-likelihood ratio <0.05). As **Table 3** shown, we discovered that only when night sleep duration is within 3.5–7.5 hours, per hour increase of night sleep bring about 15% reduced risk of developing diabetes [HR (95% CI) = 0.85 (0.77, 0.93)]. When night sleep duration exceeds 7.5 or less than 3.5, this linear association became statistically insignificant, suggesting that this linear association may be restricted in 3.5–7.5 hours of night sleep.

**Table 3:** Non-linear associations of night sleep duration with new-onset in Chinese middle-aged and older adults.

Model type	Adjusted HR (95% CI), P-value
Fitting by the standard linear model	0.93 (0.89, 0.97) **0.0018**
Fitting by the three-piecewise linear model	
Inflection point	3.5, 7.5
Night sleep hour <3.5	1.35 (1.00, 1.84) 0.0508
3.5 - 7.5	0.85 (0.77, 0.93) **0.0007**
Night sleep hour > 7.5	1.07 (0.88, 1.32) 0.4841
P for Log-likelihood ratio	**0.022**

Model were adjusted for age, sex, education, marital status, living residence, smoking status, drinking status, BMI, hypertension, and dyslipidemia, and afternoon snap duration.

Bold P values are significant at P < 0.05.

BMI, body mass index; HR, hazard ratio; CI, confidence interval.

### 3.3 Interaction and stratified analysis

**Figure 4:**
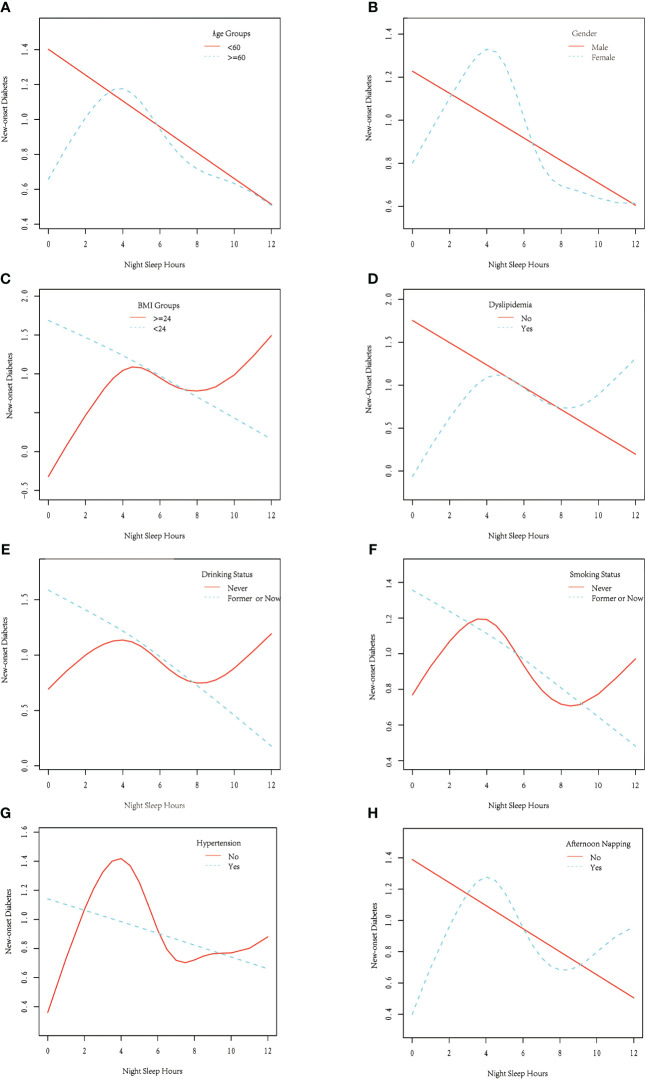
Stratified analysis of the non-linear association between night sleep duration and new-onset diabetes in Chinese middle-aged and older adults, stratified by **(A)** age, **(B)** gender, **(C)** BMI, **(D)** dyslipidemia, **(E)** drinking status, **(F)** smoking status, **(G)** hypertension and **(H)** afternoon napping (Each stratification was adjusted for age, sex, education, marital status, living residence, smoking status, drinking status, BMI, hypertension, and dyslipidemia, and afternoon snap duration, except the stratification factor itself). Blue dash line or solid red line represents the smooth curve fit between night sleep duration and the risk of new-onset diabetes for specific stratification. BMI, body mass index).

To identifying potential distinctive subgroups or potential modifiers on former identified association, we further performed interaction and stratification analysis in this section. Age, gender, BMI, smoking status, drinking status, hypertension, dyslipidemia, and afternoon napping were selected as potential modifiers. As shown in **Figure 4**, non-linear tendency only exists in some specified subgroups: age >=60, female, BMI >=24, having dyslipidemia, never drink and never smoke. **Table 4** shown that BMI (BMI >=24: HR = 0.88,95%CI: 0.82–0.93; BMI<24: HR = 0.99, 95%CI: 0.92,1.06; P for interaction = 0.0215) and dyslipidemia (have dyslipidemia: HR = 0.88,95%CI: 0.82–0.94; have no dyslipidemia: HR = 0.97, 95%CI: 0.91,1.03; P for interaction = 0.0333) might exhibit interaction effects on the association between night sleep duration and new-onset diabetes.

**Table 4:** Interaction and stratification analysis for the associations of night sleep duration (continuous) with new-onset diabetes in Chinese middle-aged and older adults.

Stratification variables	Adjusted HR (95% CI), P-value	Interaction P-value
Age		0.9499
>= 60	0.93 (0.87, 0.99) 0.0297	
<60	0.93 (0.87, 0.99) 0.0247	
Gender		0.2156
Male	0.95 (0.90, 1.00) 0.0661	
Female	0.89 (0.82, 0.96) 0.0043	
BMI		**0.0215**
>=24	0.88 (0.82, 0.93) <0.0001	
<24	0.99 (0.92, 1.06) 0.7447	
Smoking Status		0.9342
Never	0.93 (0.88, 0.98) 0.0042	
Former or Now	0.97 (0.89, 1.05) 0.3970	
Drinking Status		0.383
Never	0.95 (0.90, 1.00) 0.0469	
Former or Now	0.92 (0.85, 0.99) 0.0307	
Hypertension		0.129
Yes	0.89 (0.84, 0.96) 0.0010	
No	0.96 (0.90, 1.02) 0.2082	
Dyslipidemia		**0.0333**
Yes	0.88 (0.82, 0.94) 0.0002	
No	0.97 (0.91, 1.03) 0.3187	
Afternoon Napping		0.6355
Yes	0.95 (0.89, 1.01) 0.0943	
No	0.93 (0.87, 0.99) 0.0183	

Each stratification was adjusted for age, sex, education, marital status, living residence, smoking status, drinking status, BMI, hypertension, and dyslipidemia, and afternoon snap duration, except the stratification factor itself.

Bold P values are significant at P < 0.05.

BMI, body mass index; HR, hazard ratio; CI, confidence interval.

### 3.4 Sensitivity analyses

Three sensitivity analyses as describe in Method were proposed to further confirm our findings. As shown in **Supplementary Table 2**, no obvious difference between the sensitivity analyses and our original results (**Table 2**), Curve-fitting with addictive Cox model also presented similar variation tendency (**Supplementary Figures 1–3**).

## 4 Discussion

This 7-year longitudinal cohort study examined the relationship between night sleep duration and diabetes risk, with a focus on middle-aged to older Chinese adults. After careful adjustment for potential confounders, we discovered that a night sleep duration of no more than 8 hours significantly lowered diabetes risk. Furthermore, 7.1–8 hours of nocturnal sleep was found to be associated with the lowest incidence of diabetes. Using the addictive Cox regression approach and a piece-wise model, we identified a temporal range of 3.5–7.5 hours where the negative linear connection between night sleep and new-onset diabetes is strong. Within this interval, each hour of prolonged night sleep duration was related with a 15% reduction in the chance of acquiring diabetes over the next seven years, when compared to not increasing sleep duration. 3.5 and 7.5 hours were discovered to be two critical threshold effect cutoffs for this limited time frame. When the sleep duration exceeds 7.5 hours or under 3.5 hours, no more extra benefit or risk will follow in regard of developing diabetes. As a result, we choose to refer to it as the “restricted linear association”. In the subsequent interaction and stratified analysis, we identified some potential modifiers of this restricted linear association. Restricted linear association was observed only in subgroup with age >=60, female, BMI >=24, having dyslipidemia, never drink and never smoke. In the interaction analysis, BMI (P for interaction = 0.0215) and dyslipidemia (P for interaction = 0.0333) both exhibited significant modification effects on the association between night sleep duration and new-onset diabetes when only considering their standard linear effect. Our results remain constant after three sensitivity analyses. Overall, we proposed a novel working model that has the potential to alter the conventional view of the non-linear relationship between night sleep length and diabetes risk.

The relationship between night sleep duration and the risk of diabetes remains an intriguing study topic in recent decades. Numerous studies have revealed a distinct non-linear association between night sleep duration and diabetes, with a widely supported U-shaped association being particularly notable (4, 10, 11, 14, 21–23). This working model suggests the existence of a narrow optimal sleep interval or optimal sleep duration for the lowest risk of diabetes, beyond or below which the risk of diabetes tends to rise. Many studies have substantially verified this characteristic U-shape association (4, 11, 21–23), and 7–8 hours of night sleep was selected as the optimal sleep duration and has been extensively promoted as a specific health recommendation (24). However, other studies using a Chinese sample with a perspective design report contradictory finding. A research of 34825 people from the Shanghai Men’s Health research found that prolonged sleep duration (>= 8 hours) was linked to an increased risk of diabetes [HR(95% CI) = 1.2(1.0–1.3), when compared to the group with 7 hours of sleep] (14). Another study of 11539 Chinese participants over 3 years found that those who slept more than 9 hours per night had a higher risk of newly diagnosed type 2 diabetes. However, there was no significant difference in risk between those who slept less than 6 hours per night and those who slept 7–8 hours per night (10). A study conducted by Li, using a parallel dataset to ours, validated a U-shaped nexus between sleep duration and diabetes incidence, though the generalizability was confined primarily to the older Chinese demographic aged over 65 years (11), despite the fact that not only the older but also the middle-aged bear the majority of the burden of diabetes (25). The variations in their findings could be attributed to differences in data sources and analytic methodology. Although these studies using a Chinese population have provided useful insights into diabetes prevention, evidence from a representative sample of middle-aged and older Chinese with adequate follow-up time remains scarce.

To the best of our knowledge, this is the first study to support the longitudinal relationship between night sleep duration and diabetes risk, employing a representative sample of middle-aged and older Chinese with a 7-year follow-up period. In this study, we developed a novel working model for the effect of night sleep duration on new-onset diabetes. Instead of the U-shaped link, we discovered a restricted time window in which each hour of longer night sleep duration was related with a 15% reduction in the chance of acquiring diabetes over the next 7 years. Outside of this interval, no substantial link between the two can be established since the risk of sleep deprivation and the benefit of increased sleep duration have both become saturated. This working model is especially relevant because it may not accurately reflect the diabetes risk associated with prolonged sleep and the supposed recommended sleep length for diabetes prevention. Intriguingly, Jin’s recent work utilizing UK biobank samples demonstrated that accelerometer-measured short but not long sleep duration is related with a greater risk of incident type 2 diabetes in a 7-year follow-up, which is consistent with our findings (26).

Potential reasons for the common U-shape relationship have been gathered. Multiple validated pathways strongly link insufficient night sleep duration at baseline to the onset of diabetes, implying biological roles for insulin resistance, leptin, ghrelin, and inflammatory cytokines, as well as behavioral roles for increased energy intake and decreased decision-making ability (27). However, the causes for the link between prolonged night sleep duration and diabetes are somewhat vague. Long sleep duration may indicate low socioeconomic level, poor health, insufficient physical exercise, and psychological illness (28, 29), or it may be related with poor sleep quality (30). Furthermore, because questionnaire-based surveys are subjective, they may overestimate sleep duration. However, a UK-biobank study that used accelerometer-measured sleep duration found results that were congruent with ours, indicating that there is no significant link between extended sleep duration and diabetes risk (26). Sizhi’s mendelian randomization (MR) research revealed that genetic predictors of lengthy sleep duration are not associated with an increased risk of diabetes (31), despite the fact that MR analysis can only assess exposure effects across a life span rather than individual life stages. Compared to the traditional U-shaped model, our analysis underscores the detrimental impact of short sleep duration on diabetes risk. Additionally, we demonstrate saturation effects for both the risk associated with decreased sleep duration and the protective influence of prolonged sleep. Our model aligns with the foundational mechanisms of the U-shaped curve but distinctly emphasizes the benefits of extended sleep as a preventive strategy against diabetes. In scenarios where sleep quality cannot be enhanced, longer sleep duration may serve as an effective compensatory mechanism. Our findings suggest that maintaining an extended sleep duration habitually does not increase diabetes susceptibility.

Some potential modifiers for the suggested nonlinear connection were identified in interaction and stratified analysis. Restricted linear connection only present in several specific subgroups, including those above the age of 60, female, with a BMI greater than 24, dyslipidemia, never drinking or smoking. There are two plausible explanations for this discovery. One is the smaller sample size will result in the removal of the nonlinear relationship in some subgroups, as detecting nonlinear relationships requires a large sample size. The other explanation is the subgroup factors may have a modifying effect on this nonlinear relationship. The restricted linear intervals appeared only in select subgroups, and their locations, lengths, and slopes varied, indicating that the stratified variables may have an interaction effect. When only the strand linear impact was investigated, BMI and dyslipidemia were identified as significant modifiers, with the overall beneficial benefit of night sleep time appearing to be more evident in people with greater BMI and dyslipidemia. These could be owing to a common mechanism between short sleep duration and obesity with diabetes (32). The high prevalence of obstructive sleep apnea (OSA) in the people with obesity also contributed to the modified effects of BMI (33) and dyslipidemia (34). These findings underscored the need for clinicians and policymakers to pay more attention to extending sleep time and overall sleep quality for persons with higher BMI and dyslipidemia.

Our findings reveal a restricted linear association between night sleep duration and diabetes risk within the 3.5 to 7.5-hour range, where each additional hour of sleep correlates with a 15% reduction in the likelihood of developing diabetes. This restricted time window suggests that within this specific range, the benefits of extended sleep duration on reducing diabetes risk are significant. However, beyond this range, the protective effects of sleep become saturated, and no further significant association is observed. This phenomenon implies that both the detrimental effects of insufficient sleep and the benefits of prolonged sleep reach a plateau within this window.

The explanation for this restricted time window could be attributed to the body’s physiological and metabolic adaptations. Within the 3.5 to 7.5-hour sleep duration, the body optimally balances its restorative processes, including hormonal regulation, glucose metabolism, and inflammatory responses. Shorter sleep durations may lead to sleep deprivation, resulting in metabolic dysregulation and increased diabetes risk. On the other hand, longer sleep durations might not provide additional benefits due to a saturation effect, where the body’s capacity to benefit from extended sleep is maximized.

This study highlights the importance of maintaining an optimal sleep duration within the identified restricted time window to minimize diabetes risk. Our findings differ from the U-shaped relationship reported in other studies, suggesting a more nuanced understanding of the sleep-diabetes nexus in the Chinese population.

## 5 Limitation

The strengths of the present study include its prospective design, long follow-up duration, and precise demonstration of the non-linear association. However, this study had several limitations. Our study, for instance, did not incorporate potential confounding determinants like economic status, physical activity levels, and depressive symptomatology in the relationship matrix of sleep duration and diabetes incidence. Given our employment of the modified International Physical Activity Questionnaire-short form and the ensuing sizable missing data proportion pertaining to physical activity, the integration of such variables could have inadvertently distorted energy expenditure computations, leading to a significantly constrained sample scope. Additionally, the intricate interplay between excessive sleep, insomnia, and depression is noteworthy. While both sleep extremes can catalyze depressive states, depression can reciprocally truncate sleep spans (35–37). Thus, the sleep-diabetes nexus might be confounded or partially mediated by underlying depressive dynamics. Another potential pitfall lies in the self-reported nature of diabetes diagnosis, potentially introducing recall biases. Yet, a multitude of studies have consistently asserted the high congruence between self-reported diabetes, corroborative medical histories, or physician-validated diabetes diagnoses, boasting a specificity surpassing 90% (38–41). Besides, the lack of dietary data for CHARLS is another limitation which should be noticed. Additionally, only baseline data were used to predict future diabetes risk in this study. Future studies should account for the dynamic nature of nighttime sleep duration and time-varying confounders, employing updated longitudinal data and advanced modelling techniques to enhance causality assessments. Conclusively, it is imperative to interpret our findings with a geographical and demographic lens, principally catering to middle-aged to older populations within China and analogous middle-income nations.

## 6 Conclusion

In conclusion, a time interval was identified within which night sleep duration is linearly associated with diabetes risk, but outside of which no significant link between the two can be established because both the risk of sleep deprivation and the benefit of increased sleep duration have become saturated. Furthermore, the protective benefits of increased sleep duration on diabetes were reconfirmed and found to be modified by BMI and dyslipidemia.

This study has established a precise temporal correlation between night sleep duration and the risk of new-onset diabetes among middle-aged and older adults in a Chinese cohort, underlining a restricted linear association within a defined interval of 3.5 to 7.5 hours of nightly sleep. Beyond this range, the relationship attenuates, signaling a saturation point where both the detrimental effects of sleep deprivation and the advantageous impacts of extended sleep no longer influence diabetes risk. This phenomenon suggests a potential threshold effect, where sleep durations outside this range do not contribute additional protective benefits against diabetes.

Furthermore, our analysis reconfirmed the protective role of increased sleep duration on diabetes risk and highlighted its modification by BMI and dyslipidemia. Specifically, individuals with higher BMI and dyslipidemia exhibited a more pronounced protective effect from extended sleep duration. These findings underscore the need for tailored sleep recommendations that consider individual metabolic profiles, particularly for those at higher risk due to obesity and lipid abnormalities.

Our study contributes to the growing body of evidence on the importance of adequate sleep for metabolic health and emphasizes the necessity for diabetes and pre-diabetes interventions that promote optimal sleep duration as part of diabetes prevention strategies. Future research should explore the underlying mechanisms of this restricted linear association and investigate the potential benefits of sleep interventions in high-risk populations.

## Data availability statement

The original contributions presented in the study are included in the article/**Supplementary Material**. Further inquiries can be directed to the corresponding author.

## Ethics statement

The studies involving humans were approved by the Ethics Committee of Peking University. The studies were conducted in accordance with the local legislation and institutional requirements. The participants provided their written informed consent to participate in this study.

## Author contributions

MC: Conceptualization, Data curation, Methodology, Software, Visualization, Writing – original draft, Writing – review & editing. BL: Conceptualization, Data curation, Methodology, Software, Visualization, Writing – original draft, Writing – review & editing. YZ: Writing – original draft, Writing – review & editing. GF: Data curation, Methodology, Visualization, Writing – original draft, Writing – review & editing.
