# Supplementary material for: Restricted linear association between night sleep duration and diabetes risk in middle-aged and older adults: a 7-year follow-up analysis from the China health and retirement longitudinal study
Source: Front Endocrinol (Lausanne). 2024 Jun 20;15:1364106. doi: 10.3389/fendo.2024.1364106 (PMC11222314; doi:10.3389/fendo.2024.1364106)
Supplement: Supplementary file 1 [file DataSheet_1.docx]

**Supplemental file**

**Supplemental Table 1.** **Metabolic markers of the analyzed participants grouped by new-onset diabetes**

| Characteristics | |  | | Having new-onset diabetes | | | | |  | |
| --- | --- | --- | --- | --- | --- | --- | --- | --- | --- | --- |
|  |  | Overall | | No | | Yes | P-value | | | |
| HbA1c percentage (mean (SD)) | | 5.10 (0.39) | | 5.09 (0.38) | | 5.24 (0.44) | | | <0.001 | |
| FGu mg/dl (mean (SD)) | | 100.05 (11.76) | | 99.56 (11.57) | | 105.02 (12.45) | | | <0.001 | |
| TC mg/dl (mean (SD)) | | 192.80 (37.42) | | 192.34 (37.38) | | 197.46 (37.55) | | | 0.004 | |
| HDL mg/dl (mean (SD)) | | 52.24 (15.07) | | 52.55 (15.04) | | 49.06 (15.03) | | | <0.001 | |
| LDL mg/dl (mean (SD)) | | 117.40 (33.92) | | 117.10 (33.86) | | 120.51 (34.42) | | | 0.034 | |
| TG mg/dl (mean (SD)) | | 121.31 (74.17) | | 119.32 (72.47) | | 141.47 (87.12) | | | <0.001 | |

Data are presented as mean (SD) or n (%).

Abbreviations: HbA1c: glycosylated hemoglobin; FGu: fasting glucose; TC: total cholesterol; HDL: high-density lipoprotein; LDL: low-density lipoprotein; TG: triglyceride; SD: standard deviation.

**Supplemental Table 2. Sensitivity analyses f****or the associations of night sleep duration (discontinuous) with new-onset diabetes in Chinese middle-aged and older adults**

| Characteristic | N | Event N | HR | 95% CI | P-value |
| --- | --- | --- | --- | --- | --- |
| **Sensitivity Analysis 1: Additionally Adjusting Sleep Quality** | | | | | |
| Night sleep duration | |  |  |  |  |
| <=5 | 1,600 | 187 | Reference | |  |
| >8 | 439 | 38 | 0.82 | 0.57, 1.18 | 0.3 |
| 5.1-6 | 1,132 | 94 | 0.77 | 0.59, 1.00 | **0.046** |
| 6.1-7 | 1,050 | 84 | 0.76 | 0.58, 1.01 | 0.062 |
| 7.1-8 | 1,186 | 82 | 0.67 | 0.50, 0.89 | **0.006** |
| **Sensitivity Analysis 2: Alternatively Adjusting Drinking Amount** | | | | | |
| Night sleep duration | |  |  |  |  |
| <=5 | 1,610 | 188 | Reference | |  |
| >8 | 441 | 38 | 0.71 | 0.50, 1.00 | 0.052 |
| 5.1-6 | 1,137 | 94 | 0.71 | 0.55, 0.91 | **0.007** |
| 6.1-7 | 1,055 | 85 | 0.68 | 0.53, 0.89 | **0.004** |
| 7.1-8 | 1,194 | 83 | 0.58 | 0.45, 0.75 | **<0.001** |
| **Sensitivity Analysis 3*: Pooled Result from 5 Imputed Datasets** | | | | | |
| Night sleep duration | |  |  |  |  |
| <=5 | 1,847 | 214 | Reference |  |  |
| >8 | 504 | 38 | 0.63 | 0.45, 0.90 | **0.01** |
| 5.1-6 | 1,293 | 103 | 0.68 | 0.54, 0.87 | **0.002** |
| 6.1-7 | 1,186 | 93 | 0.67 | 0.52, 0.86 | **0.001** |
| 7.1-8 | 1,354 | 101 | 0.63 | 0.50, 0.80 | **<0.001** |

Sensitivity Analysis 1: The model was adjusted for age, sex, education, marriage, living residence, smoking status, drinking status, BMI, hypertension, and dyslipidemia, afternoon snap duration, as well as sleep quality.

Sensitivity Analysis 2: The model was adjusted for age, sex, education, marriage, living residence, smoking status, BMI, hypertension, and dyslipidemia, afternoon snap duration, as well as drinking amount.

Sensitivity Analysis 3: The model was adjusted for age, sex, education, marriage, living residence, smoking status, drinking status, BMI, hypertension, and dyslipidemia, and afternoon snap duration. *Regression coefficients from the results of 5 imputed datasets were pooled based on Rubin’s rules.

Bold P values are significant at P < 0.05.

Abbreviations: BMI: body mass index; HR: hazard ratio; CI: confidence interval.


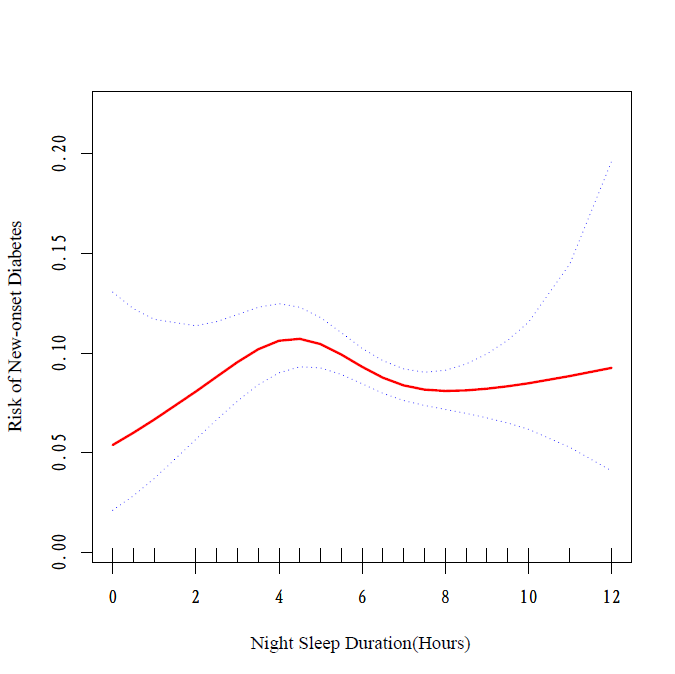


Supplemental Figure 1. Sensitivity Analysis 1 for the non-linear associations of night sleep duration with new-onset diabetes in Chinese middle-aged and older adults. The model was adjusted for age, sex, education, marriage, living residence, smoking status, drinking status, BMI, hypertension, and dyslipidemia, afternoon snap duration, as well as **sleep quality**.

Abbreviations: BMI: body mass index; HR: hazard ratio; CI: confidence interval.


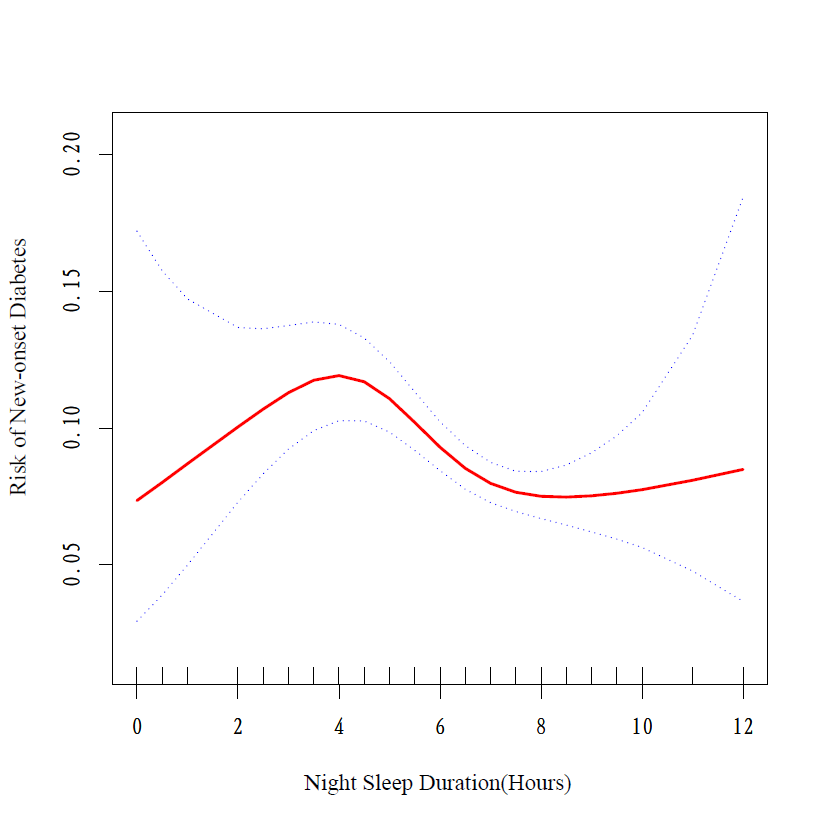


Supplemental Figure 2. Sensitivity Analysis 2 for the non-linear associations of night sleep duration with new-onset diabetes in Chinese middle-aged and older adults. The model was adjusted for age, sex, education, marriage, living residence, smoking status, BMI, hypertension, and dyslipidemia, afternoon snap duration, as well as **drinking amount**.

Abbreviations: BMI: body mass index; HR: hazard ratio; CI: confidence interval.


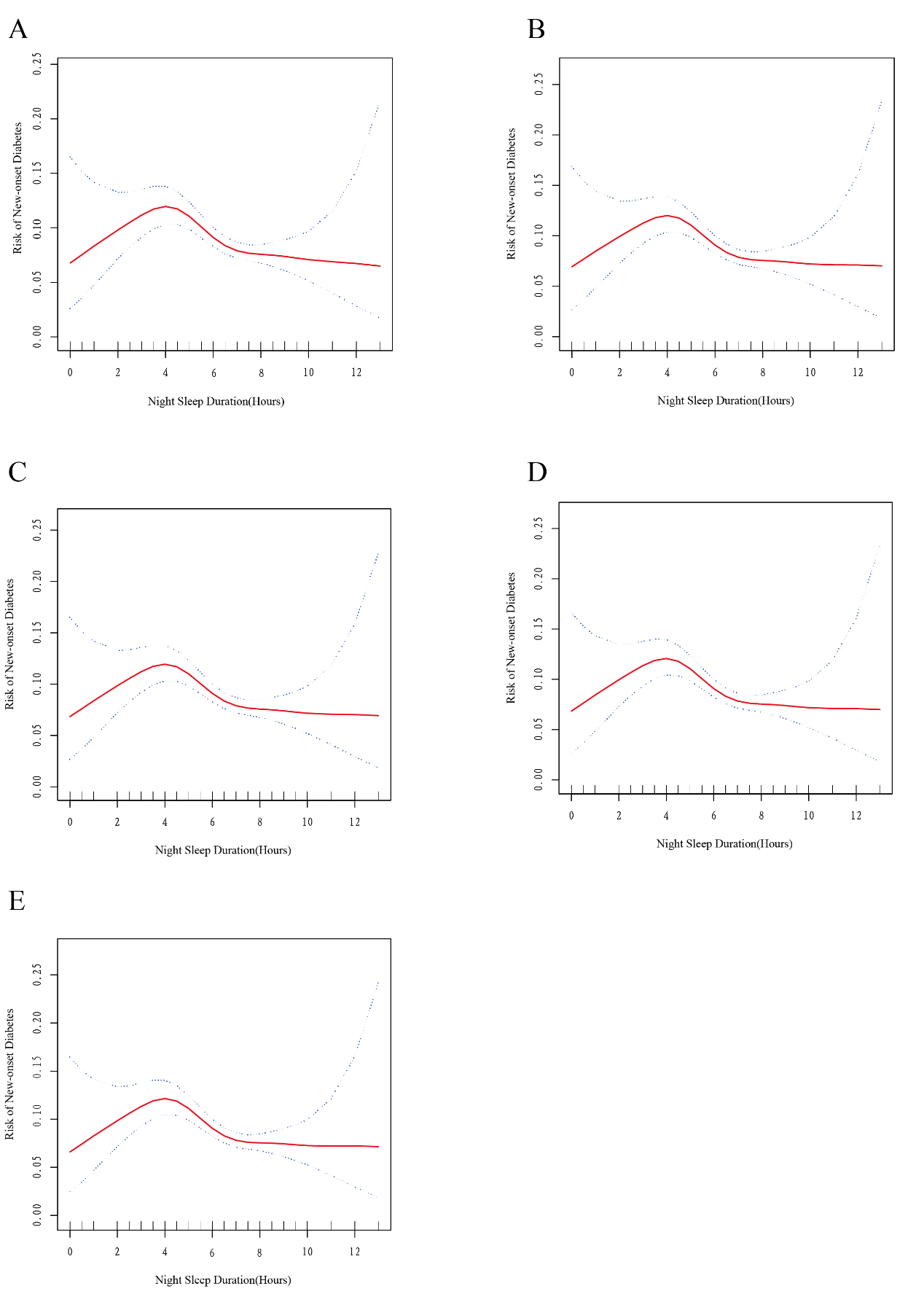


Supplemental Figure 3. Sensitivity Analysis 3 for the non-linear associations of night sleep duration and afternoon napping with new-onset diabetes in Chinese middle-aged and older adults. The model was adjusted for age, sex, education, marriage, living residence, smoking status, drinking status, BMI, hypertension, and dyslipidemia, and afternoon snap duration. Five imputed datasets were generated by multiple imputation method with R package ‘mice’, and each imputed dataset was used for generating graphics of smooth curve fittings.
